# Improved professional practices in social services through Emotional Labor strategies

**DOI:** 10.3389/fpsyg.2023.1145175

**Published:** 2023-04-17

**Authors:** Tea Gvelesiani, Shorena Sadzaglishvili, Ketevan Gigineishvili, Ketevan Lekishvili, Salome Namitcheishvili

**Affiliations:** ^1^GIPA – Georgian Institute of Public Affairs, Tbilisi, Georgia; ^2^School of Arts and Sciences, Ilia State University, Tbilisi, Georgia; ^3^Georgian Association of Social Workers, Tbilisi, Georgia; ^4^The Department of Sociology and Social Work, Iv. Javakhishvili Tbilisi State University, Tbilisi, Georgia

**Keywords:** Emotional Labor, social work, burnout, professional growth, Georgia

## Abstract

The present study provides an analysis of Emotional Labor (EL) and its consequences for professional social work practitioners in Georgia. This mixed-methods study comprised two stages. First, a qualitative study was conducted to determine the organizational characteristics defined by social work practitioners (*N* = 70). Second, a quantitative study was undertaken among the members of the Georgian Association of Social Workers (*N* = 165) to determine the direct and indirect influences of organizational characteristics on EL and work outcomes, namely, personal accomplishment and burnout. The results are pragmatic and applicable for organizations providing social services to gain positive results at the individual and organizational levels.

## Introduction

Social work as a profession aims to enhance human wellbeing and assist people in need to meet their basic and complex needs (IFSW, 2014). Social work practitioners are service providers who mostly need to perform Emotional Labor (EL) in addition to Intellectual Labor. According to Grandey (2000), EL is “the process of regulating both feelings and expressions for organizational goals”. The term “Emotional Labor” was first established by Hochschild (2003) who explained it as “the management of feelings to create a publicly observable facial and bodily display; Emotional Labor is sold for a wage and therefore has exchange value” (p. 7). The employees have to make sincere efforts to practice and adhere to the organization's emotional display rules (Zapf and Holz, 2006; Johnson and Spector, 2007). Generally, employees are expected to be polite, warm, and friendly to internal and external stakeholders while expressions of anger and frustration are strongly discouraged (Smollan, 2006; Sisley and Smollan, 2012).

Hochschild (1983) definition of EL presumes that service providers attempt to manage their emotions either by engaging in Surface Acting (employees modify their displays without shaping inner feelings) or Deep Acting (employees modify internal feelings to be consistent with display rules) (Kariou et al., 2021). Ashforth and Humphrey (1993) defined EL as expressing socially desired emotions during service transactions. For instance, a service agent might naturally feel what he or she should express without stirring up emotions discussed by Hochschild (Yang and Chen, 2021). In addition, several researchers have identified social workers to have a social role in caring, focusing on prevention, sharing knowledge, listening to people, and showing commitment and emotional support to clients (Leidner, 1991; Dubayle et al., 1993; Moesby-Jensen and Nielsen, 2015).

Emotional demands are more frequent in jobs that involve relational work, and the management of social workers' emotions has been gradually recognized as a crucial element of relational client work over the decades (Turtiainen et al., 2020). Given that the profession of social work is one of the occupational categories high in EL, different measures have been discussed in social work literature to provide support to social workers to manage their emotional work. Some of the measures include social work supervision, individual counseling, and guidance on self-care (Global Social Service Workforce Alliance, 2020). The social work supervision system is considered one of the effective mechanisms that provide support to social workers. Supervision is viewed as a key means of helping staff do a difficult job and of promoting quality in social service work. Supervision in social services is a supportive relationship. It is carried out in regular meetings that focus on accountability, wellbeing, and skill development. Practice-based evidence and research have shown that structured, supportive, and reflective supervision helps to improve worker retention and performance, prevent burnout and results in a higher quality of services, and support to children and families (Global Social Service Workforce Alliance, 2020).

Supervision is necessary to support social service workers, but it is not always fully sufficient to meet staff support needs. Supervision alone cannot meet all the needs of the staff's wellbeing, and other forms of helping measures such as individual confidential counseling and guidance on self-care should be made available. Guidance on self-care should include advice on how staff can take care of themselves physically and mentally to maintain a positive attitude toward work and positively manage work tasks and relationships to better manage normal stress, prevent negative stress, and maintain a positive work-life balance (Global Social Service Workforce Alliance, 2020).

## Social service workforce in Georgia

In Georgia, social work as a profession has a history of over 20 years. It began in 1999 with the first professional training in social work initiated by the Ministry of Education and Science with technical support from UNICEF-Georgia and international NGOs (Sadzaglishvili, 2017). The first batch of social workers were childcare workers responsible for providing alternative services to children and their families including the reintegration of children with their biological families as well as developing kinship care, foster care, small group homes, and preventive services. Gradually, social workers came to be involved in the fields of health/mental health, the criminal justice system, disability, and school social work. Currently, social workers are engaged in micro, mezzo, and macro levels, and their responsibilities differ depending on which level they are working. For instance, social workers at the micro-level typically conduct bio-psycho-social assessments and interventions using individual and family counseling skills while mezzo-level social workers work with communities to improve the functioning of the community, and macro-level social workers are involved in research, advocacy, and formulation of social/health/mental health policies (Sadzaglishvili, 2017).

The Georgian Association of Social Workers (GASW) has played a critical role in developing the social work profession in Georgia. It is the largest membership organization of professional social workers in Georgia with over 700 members. The GASW has been instrumental in developing social work practice, education, and a regulatory framework for Georgia (Partskhalaladze et al., 2020). It is also a part of the global social work professional community (GASW, 2022).

GASW was established in 2004 by the first group of American-educated social workers. It was set up as a local non-governmental organization (NGO) to support the continued development of the profession within Georgia. Besides providing professional expertise and support to the Government of Georgia, social service providers, and social workers, GASW has been active in establishing a strong educational framework for social work as a discipline based on recognized professional standards. In particular, GASW was instrumental in establishing social work academic programs at the state universities in 2006. As a result, Bachelor, Master, and Doctoral programs were established in social work to prepare professionals in this field (Sadzaglishvili, 2017); currently, these academic programs have ~672 graduates, most of whom are members of the GASW. In addition, there are ~561 “certified social workers” who have acquired very basic academic knowledge through short-term training courses and are eligible to fulfill the role of social workers, especially in the regions of Georgia (GASW, 2022).

Over the past decade and a half, Georgia has been engaged in social assistance, child welfare, and justice system reforms and these measures have strongly impacted the development of the social service workforce. Key issues that are being addressed by the social service workforce include the deinstitutionalization of the childcare system, development of family support and family substitute services, prevention of violence against children, discrimination against representatives of ethnic minority groups and migrants, social exclusion of persons with disability, and supporting families who are experiencing poverty, especially, in remote mountainous areas. The introduction of the new Law of Georgia on Social Work (2018) is a critical milestone in the development and strengthening of social workers in the country. The abovementioned introduction of social reforms being reflected in the new law has provided fresh impetus to the workforce development of social workers. The Law on Social Work also provides for the protection of the title of a social worker by stipulating that only those with a formal education in social work (e.g., a BA or MA) have the right to be employed as social workers. To prevent a workforce-related crisis, the law offers a mechanism whereby individuals without a formal education in social work but currently employed in social worker positions (or have acquired at least 1 year of prior experience working as a social worker) can obtain the authority to practice as social workers by completing a certification process (Law of Georgia on Social Work, 2018 Article 44, 3). The law also defines the certification process as a temporary measure offered only during an interim period, after which, according to the law, only individuals with social work degrees would be authorized for employment in social worker positions (Namitcheishvili and Rogers, 2018). The certification process includes the completion of a 30–45 credit training course delivered over a short period of time and is envisaged as an interim measure to prepare a pool of social workers to meet the employment demands of the country. However, there are no specified standards set for the training course and/or the accreditation processes (GASW, 2022). On the one hand, the social service workforce is moving toward full professionalization with the introduction of the new Law on Social Work, which effectively protects the title of “social worker” by establishing that a social worker must have a qualifying education or an equivalent certification. In due time, there will no longer be uncertified social workers in Georgia, and all personnel with the title “social worker” will have a social work degree or equivalent certification confirming their competency in social work (Namitcheishvili and Rogers, 2018). However, on the other hand, no quality standards or regulations have been put in place that ensure the quality of the certificate course, which jeopardizes the professionalization of the social work workforce and its development (GASW, 2022).

Since 2020, GASW has been actively involved in the process of strengthening the capacity of the State Care Agency, which is one of the biggest service providers as well as employers of social workers with support from UNICEF and the EU. Social workers were provided with training in case management, with a particular emphasis on enhancing social work skills to deal with child abuse and child neglect cases, as well as to identify, assess, and prevent the risk of suicide in children and adolescents. GASW also developed and conducted a training module for implementing safety standards in the State Care Agency, which was meant for all the social workers at the Agency. The training included topics such as organizational culture and its impact on work ethic, safety at the workplace, professional burnout and its prevention, and briefings on the procedures and distribution of responsibilities among the agency staff aimed at meeting safety standards. In addition, GASW has supported the State Care Agency in introducing social work supervision system and building the capacity of social work supervisors.

A recent GASW study reported that the social workers employed by the State Care Agency found their work emotionally exhaustive as they had to function in stressful and complex situations (GASW, 2022). In particular, the GASW study revealed that social workers felt that they worked 24/7, though this is their perception rather than a reality as the total number of working hours per month is according to the hours allowed by the labor laws of the country. In addition, the report also revealed several other factors impacting the social workers: (1) They personally became aware of the effects of severe poverty, loss, violence, or discrimination on individuals known to them. They were impacted by the suffering of others every day in their work and found it difficult to manage their own feelings, even while they sought to provide practical assistance, guidance, and advocacy for others; (2) Often they were left alone with their clients as the accessible and diverse family support services were limited to meet the needs of the clients; (3) Furthermore, they faced their own challenges, such as low remuneration, or lacked resources to support their work such as computers, data, transport, or secure meeting spaces for the clients to respect their confidentiality; (4) Social workers often worked in multi-professional environments where, at times, other professionals did not understand their role and mandate (given that the concept of social work practice is relatively new in Georgia) and were required to advocate for themselves and for their clients. Considering these factors, social workers found their work emotionally highly demanding and pressing and felt professionally diminished (GASW, 2022).

It should be noted that in the process of supporting the State Care Agency workforce, GASW used a participatory approach. All processes included the social work practitioners, the professional association, the professional union, and experts and organizations working on human rights. GASW regularly collects data on its members' professional development and job satisfaction to plan activities for them in the future.

## Theoretical framework of Emotional Labor

The concept of emotion is generally related to the cognitive assessment of a situation and physiological excitement (Grandey, 2000, p. 98). Emotion is a short-term condition (fear, rage, joy, and anger), which is associated with a particular stimulus/irritant. Mood, on the other hand, is more diffused and less connected with any particular stimulus (Frijda, 1993). While emotions are related to a specific behavior, the same cannot be said about mood. The term “affect” is more general and includes both emotion and mood. Emotions can cause a specific behavior (escape, quarrel, hugs) and can have a direct or indirect influence on a person's physiological, cognitive, and social processes.

The theory of emotion regulation by Gross (1998) can be most accurately applied to the mechanism of EL performance (Grandey, 2000). It is defined as “the processes by which individuals influence which emotions they have, when they have them, and how they experience and express these emotions” (Gross, 1998, p. 275), and provides a beneficial guiding framework for EL. According to the emotion regulation theory, the individual can regulate emotions at two points: (1) antecedent-focused emotion regulation, where the individual modifies the situation or the perception of the situation to adjust his or her emotions, and (2) response-focused emotion regulation, when the person tends to an emotional response but manipulates how he or she expresses that emotional response by “directly influencing physiological, experiential, or behavioral responding” (Gross, 1998, p. 285; Yang and Chen, 2021).

Conservation of Resources (COR) theory assumes that individuals make an effort to obtain, retain, and protect resources, especially resources that they value and are central to fulfilling their core needs and objectives (Hobfoll, 1989). According to COR theory, stress is experienced after negative events if resources have been threatened, lost, and/or not gained after the significant previous investment. The theory suggests that the physical, mental, and emotional wellbeing of people are resources exposed to loss or depletion by the stress of performing EL. That is, EL leads to the loss of resources and has various negative outcomes such as burnout, negative affect, and low level of performance (Yang and Chen, 2021).

The study of EL has also been discussed by various researchers through the prism of resource conservation theory (Brotheridge and Lee, 2002; Goldberg and Grandey, 2007; Grandey and Gabriel, 2015; Huang et al., 2015). The form of Surface Acting in EL is related to emotional dissonance and requires resources to overcome existing emotions and to express another emotion, i.e., the individual loses resources in the process. It is associated with stress and stress with Emotional Exhaustion, among others. EL with Deep Acting also requires resources to modify current experienced emotion into desired emotion, but it is an invested resource that results in substituting it with customer satisfaction, positive feedback from management, and self-confidence, which relates to the effectiveness of work, etc. EL with Deep Acting is not connected to stress according to the resources conservation theory (Park et al., 2014). Based on this theory, the Surface Acting strategy for performing EL is linked to stress and Emotional Exhaustion, while the result of EL adopting Deep Acting and Genuine Acting strategies can be a source of wellbeing or job satisfaction.

Social Exchange Theory is considered among the most influential conceptual paradigms for explaining behavior in the work environment. It is derived from classical anthropological studies (e.g., Malinowski 1922, 1932 in Mauss, 1967), according to which the exchange is presented in terminologies related to economic and social values. In terms of economical exchange, there is profit and loss between the parties. Social exchange relationships develop when employers “care for the employees”, and this helps to get the desired/useful results (Cropanzano et al., 2001). In other words, social exchange relationships are mediators or interventional variables for a beneficial and fair transaction, and this relationship contributes to employees' effective working behavior and positive attitude. This cause-and-effect relationship has attracted much attention, the majority of which uses Blau's (1964) conceptual framework for the definition of social exchange relationships. Social exchange is a more behavior-oriented construction, both observable and concrete than general feelings. According to this theory, the service personnel who perform EL through Deep Acting establish simpler and higher-quality exchange relationships than the personnel who use Surface Acting to perform EL. Surface Acting contributes to social and emotional relationships becoming distant (Graen and Uhl-Bien, 1995).

The Self-Determination Theory (Ryan and Deci, 2000) is a meta-theory for motivation and personal development. This theory places on a single continuum the internal (interest, curiosity, values) and external (reward, benefit, fear of assessing other people) resources for motivation along with the social and cultural factors that influence human voluntary action and initiative. According to this theory, people have basic psychological needs such as competence, relatedness, and autonomy. If these three requirements are met, then they are motivated, have a feeling of attribution and realization of their potential and functions, and develop optimally. If the abovementioned three requirements are not met, then a person is demotivated, feels rejected, and functions inefficiently (Ryan and Deci, 2000). In this context, the social and cultural environment plays an important role in satisfying basic psychological needs.

The categories of the self-determination continuum correspond to some of the forms of EL: Surface Acting, Deep Acting, and Genuine Acting (Sisley and Smollan, 2012).

“Job Burnout”, according to the most widely accepted definition, is “the syndrome of Emotional Exhaustion, Depersonalization, and reduced Personal Accomplishment that can be experienced in individuals who have intensive relationships with people” (Maslach et al., 1997).

Despite the diversity and chaos of explanations surrounding the concept of “Job Burnout”, researchers (Freudenberger, 1974; Maslach, 1982; Schaufeli et al., 1993) agree that “Burnout”

- emerges at the individual level;
- is an internal psychological condition, which includes emotions, moods, motives, and expectations;
- is perceived by the person as a negative experience;
- is related to problems, stress, discomfort, and complication of function;
- manifests as with symptoms in people with no psychopathological experience and healthy people;
- brings negative results; and
- reduces the performance effectiveness of the work.

According to Maslach (1982), “Job Burnout” has three dimensions: Emotional Exhaustion, Depersonalization, and reduced Personal Accomplishment. Emotional exhaustion is a condition when the person experiences the exhaustion of personal emotional resources, is very vulnerable to stressors, and has a feeling of constant fatigue, all stemming from the specificity of the service. Depersonalization is a condition characterized by cynicism and distancing from others. For example, service personnel struggling with Depersonalization are distinguished by their cynical attitude toward customers and their working environment. Reduced Personal Accomplishment is a condition when the person develops feelings of incompetence and failure, and provides a lower self-estimate of their work compared to others (Maslach and Jackson, 1981; Maslach et al., 1996, 1997).

There are different theories that suggest the sequence of development of these dimensions. According to Golembiewski's phase model, the first phase of job burnout is Depersonalization, followed by reduced Personal Accomplishment, and then, Emotional Exhaustion develops (Golembiewski, 1989). However, Maslach and Leiter (2008) believe in the opposite sequence, i.e., first Emotional Exhaustion develops, followed by Depersonalization and lastly reduced Personal Accomplishment (Maslach and Leiter, 2008). Based on their research, the authors also indicated that these three dimensions could develop and process in parallel since they are reactions to different factors of the working environment (Maslach and Leiter, 2008). In the current study, we have relied on the definition of job burnout and its three dimensions as proposed by Maslach and Leiter (2008).

Psychologists have often associated emotions and emotion management with health problems (Gross, 1989, 1998; Pennebaker et al., 1990; Steptoe, 1993; Grandey, 2000). In general, individuals have the tendency to spontaneously behave in response to the stimulus of emotions, which is a kind of defense mechanism to adapt to the environment. However, in modern-day reality, a spontaneous reaction may be unrefined to the situation. Therefore, a modern adult learns to manage emotions, suppresses the existing emotion if it is unacceptable in the social context, and expresses an emotion that is suitable for the situation, though this degrades behavior activity and increases the activity of the autonomic nervous system: Retention of negative emotions reduces the immune system (Gross, 1989, 1998; Pennebaker et al., 1990; Grandey, 2000).

Performing EL by adopting a Surface Acting strategy results in the suppression of emotions, which creates emotional dissonance and is detrimental to both the organization and its clients. As already mentioned above, it is also harmful to the social worker's health.

Several studies on the relationships between Emotional Labor and burnout have been based on “the dissonance theory of Emotional Labor” (Jeung et al., 2018).

According to this theory, emotional dissonance is considered a cornerstone of Emotional Labor. It is conceptualized as a conflict between felt and displayed emotions, encompassing both potential and actually manifested emotions. It was found that employees gradually begin to experience burnout when their capacity for emotional dissonance is exhausted as a result of Emotional Labor. These studies also suggested that emotional dissonance was positively associated with burnout. In particular, employees were depleted of energy and became exhausted if they were continuously exposed to situations requiring emotional regulation, e.g., adherence to excessive display rules (Jeung et al., 2018).

According to some studies, emotional dissonance significantly predicts Depersonalization and Emotional Exhaustion (Cote and Morgan, 2002; Heuven and Bakker, 2003; Thisera, 2017). For instance, suppression of emotions leads to work dissatisfaction, which increases the tendency to quit a job.

Though various studies have explored the relationships between EL, Surface Acting, emotional dissonance, and burnout, there is less research and evidence regarding the relationship between Genuine Acting and burnout.

Job characteristics are defined as specific aspects of work, such as knowledge, skills, intellectual and physical requirements, and working conditions that can be perceived, defined, and evaluated. They are also called working factors. Job characteristics affect an employee's attitude toward labor and also the effectiveness of work performance.

According to Morris and Feldman (1996), high levels of autonomy reduced the level of emotional dissonance, i.e., personnel who were more autonomous were less likely to apply Surface Acting (faking emotions) to clients. Schneider and Brown also indicated the important role of social support in the context of EL; in particular, they found that high levels of emotional support reduced the frequency of EL through Surface Acting and promoted a positive outcome and job satisfaction (Schneider and Bowen, 1985). Therefore, the influence of organizational factors on the relationship between EL and the consequences of job performance needs to be researched.

## The current study

The current study aimed to fill some of the research gaps identified above and collect empirical evidence to determine the relationship between EL performance and social workers' wellbeing in Georgia. In existing research, EL is presented mostly as two-dimensional or something that is performed using two strategies: Surface Acting and Deep Acting. In this study, EL is presented as a three-dimensional construct adding naturally felt emotion as Genuine Acting. We assume that social workers mostly perform EL with a Genuine Acting strategy; therefore, it would be interesting to explore how this affects their wellbeing and how organizational factors influence the relationship between EL and Personal Accomplishment or burnout. Organizational factors can be controlled and changed intentionally by a management decision. Therefore, the results from this study can support organizations to make necessary changes to improve service performance while addressing social workers' wellbeing at the same time.

Our mixed-methods study comprised two stages. In the first stage, a qualitative study was conducted to determine the organizational characteristics that might be related to EL performed by professional social workers. For this reason, focus groups with social workers employed by the State Care Agency were conducted. The State Care Agency is one of the main agencies that employ social workers in Georgia in a range of services ranging from child protection to providing services for older adults and people with disabilities. In the second stage of the study, a quantitative study which examined how EL strategies affected social workers' wellbeing was conducted. The direct and indirect influences of organizational characteristics on EL and the work outcomes that could be positive (Personal Accomplishment) or negative (burnout) among professional social workers were analyzed. In the current study, EL is presented in three dimensions: Surface Acting, Deep Acting, and Genuine Acting (naturally felt emotions). Negative work outcomes such as burnout were expressed as Emotional Exhaustion and/or Depersonalization and/or reduced Personal Accomplishment.

Organizational factors that were examined in relation to EL and work outcomes were as follows: professional growth and development, social relations at work, feedback from the managers, recognition from the agency, and salary. These factors were identified as important by social workers through focus group interviews.

Our assumptions were as follows:

1. EL dimensions will predict burnout dimensions. For instance, Surface Acting will predict all three expressions of burnout, and deep and Genuine Acting will not predict burnout; Genuine Acting and Deep Acting will predict personnel accomplishment (the opposite dimension of reduced personnel accomplishment)
2. Organizational factors will have a moderating effect to minimize negative outcomes and support positive outcomes of EL.

## Materials and methods

### Participants and procedure

#### Ethics and confidentiality

The study's ethics approval (GASW-E-01.01.2022) was obtained from the Georgian Association of Social Workers' Ethics Committee.

#### Qualitative study: Data collection procedures and respondents

State Care Agency social workers were selected randomly from the six big cities of Georgia (Tbilisi, Rustavi, Telavi, Batumi, Zugdidi, and Kutaisi) where GASW had conducted a study to assess factors contributing to social workers' burnout and identify important organizational factors that could support their work performance and wellbeing.

In the qualitative study, 70 social workers (66 women and four men, with a mean age of 34.15) participated in 12 focus groups from six cities (Tbilisi, Rustavi, Batumi, Kutaisi, Zugdidi, Telavi) in Georgia. The group members were informed about the confidentiality issues.

#### Quantitative study: Data collection procedures and respondents

Data were collected *via* an electronic self-report survey from a GASW members' sample of 168 participants from 12 January to 24 March 2022. Participants were recruited *via* e-mail and other electronic means of communication. Convenience sampling was used, and all members of GASW were eligible to participate. To minimize participant dropout, the electronic survey link was first piloted, and the results were taken into consideration. The link was forwarded with a brief description of the goal of the study and instructions for completion. The potential participants were informed about the anonymity of the survey, the approximate time (20–25 min) needed to complete the questionnaire, and the criteria for participation, which entailed being a GASW member.

In the quantitative study, the mean age of the participants was 35.50 (SD = 15.37), with the sample consisting of 168 GASW members (6% men and 94% women), 55% of the participants were from Tbilisi and 45% were from other cities. Regarding the educational level, 46.6% had MSW degrees, 28% had certificates in social work, 18.5% had BSW degrees, and 7.1% had other degrees in psychology and related fields. Regarding the level of engagement, 22% worked at a macro level, 29.8% worked at the mezzo level, and 48.2% at the micro level. GASW members spent about 20 (SD = 23) h/week with their clients/beneficiaries.

### Research concept/operationalization of the variables

The research model examined the links between EL and job burnout and the indirect influence of job characteristics on these relationships. EL, according to our research, was explained as the process of the regulation and expression of emotions relevant to job demands and the situation to satisfy the client.

**Figure 1:**
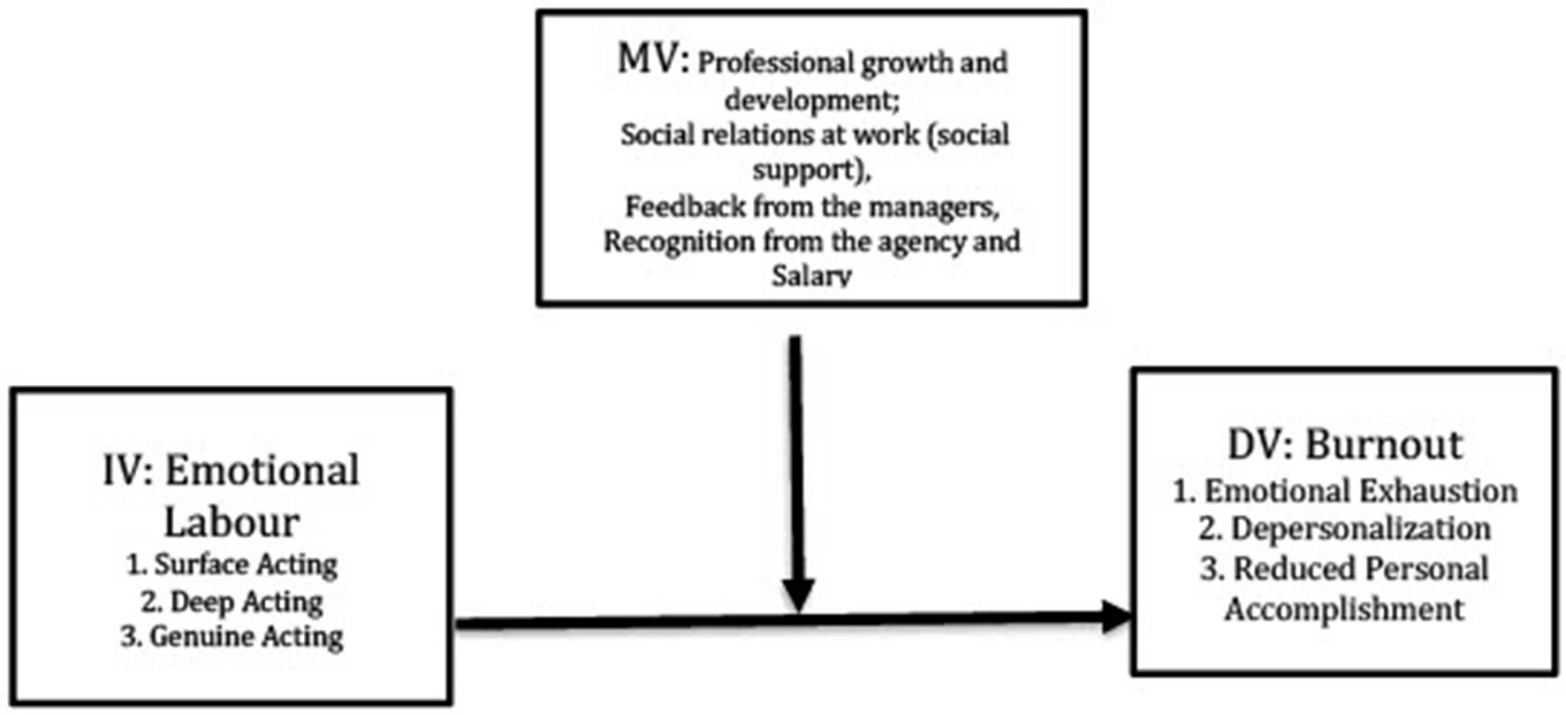
Research model: EL impact on social workers' burnout.

In our study, EL was presented in a three-dimensional (Ashforth and Humphrey, 1993) configuration: Surface Acting, Deep Acting, and Genuine Acting, all of which were regarded as predictors/independent variables (Independent Variable—IV). Burnout and Personal Accomplishment were considered as outcomes/dependent variables (Dependent Variable—DV), whereas satisfaction regarding organizational factors, such as professional growth and development, social relations at work, feedback from the managers, recognition from the agency, and salary were envisaged as moderating variables (MV). See Figure 1 for the research model.

Based on the above-discussed theories and research results regarding Emotional Labor, the main questions that we aimed to answer were:

- Which “Emotional Labor Strategy” predicts burnout for social workers?
- Can organizational factors reduce the relationship between Emotional Labor strategies and burnout dimensions?
- Can Emotional Labor strategies predict positive outcomes like Personal Accomplishment?

To answer the main questions of the study, the following hypotheses (H) were examined:

H 1. —Surface Acting of EL will positively predict Emotional Exhaustion, Depersonalization, and reduced Personnel Accomplishment; Genuine Acting and Deep Acting will positively predict Personal Accomplishment;
H 2. —Organizational factors will have a moderating effect between Surface Acting and Emotional Exhaustion and Depersonalization;
H 3. —Organizational factors will enhance the moderation effect between Genuine Acting and Personal Accomplishment and between Deep Acting and Personal Accomplishment.

### Measures

The quantitative study link encompassed a self-report inventory of (1) an adapted version of the Emotional Labor Questionnaire (Gvelesiani, 2019) and (2) Burnout (Christina Maslach, Job Burnout Inventory, MBI-GS) as well as questions on demographics and work-related variables. Data gathered on participant demographics included information on individual and workplace characteristics including age, gender, education, employment level (micro, mezzo, and macro), workload (hours spent per week in personal contact with clients), level of satisfaction with professional growth and development, social relations at work, feedback from the managers, satisfaction with salary, and recognition from the agency.

For the quantitative research instrument, questionnaires were selected based on the following criteria: (1) their theoretical basis corresponding to our research concept; (2) their adaptability to the Georgian population; and (3) their high reliability and validity.

(1) Adapted version of Emotional Labor Questionnaire

The Emotional Labor Questionnaire (Kruml and Geddes, 2000) was adapted by the Georgian psychologist Gvelesiani (2019) for her dissertation study.

The Emotional Labor Questionnaire is a 12-item self-report inventory with a 5-point Likert Scale, where 1 corresponds to never and 5 to always. Reliability indicators of the calculated scales of the Georgian version within the original test and our selection (*N* = 168) (internal consistency, Cronbach alpha coefficient) are presented in Table 1.

**Table 1:** Reliability of Emotional Labor scale.

**Scale**	**Original version of test's Cronbach's α**	**Georgian version of test's Cronbach's α**
Surface acting	0.66	0.72
Genuine acting	0.68	0.77
Deep acting	0.72	0.67

Three subscales of Emotional Labor such as **Deep Acting** (I express genuine goodwill to the beneficiary; I acknowledge my role and the emotions that beneficiaries are expecting from me; I genuinely feel compassion toward the beneficiary; I experience emotions that I must display; I make an effort to actually feel the emotions that I need to display toward others; I enjoy communication with the beneficiary), **Surface Acting** (I try to change my actual feelings to match those that I must express to the beneficiary; I just pretend to have the emotions I need to display for my job; The emotions I show to the beneficiary does not match what I truly feel at the moment), and **Genuine Acting** (I try to imagine someone close to me instead of the beneficiary to feel compassion; I put on a “mask” in order to express the right emotions for the job; I need to hide my real emotions) were maintained after completion of Confirmatory Factor Analysis (CFA) with the following fit indices: χ2 = 555.934, df = 66, *p* = 0.00.

(2) Christina Maslach, Job Burnout Inventory (MBI-GS)

The questionnaire consists of 22 statements and serves to evaluate the emotional state of the workers. MBI is designed to evaluate three components of Emotional Exhaustion syndrome, namely, Emotional Exhaustion, Depersonalization, and reduced Personal Accomplishment. Filling the MBI-GS requires 5–10 min and is a self-report inventory. Nine statements of the Emotional Exhaustion subscale measure the state when a person is exhausted and emotionally tired of work. Five statements of the Depersonalization subscale assess the state of a person, which reflects the individual's inhuman and biased feedback. High scores on both subscales indicate the severity of the state of burnout. The third subscale, which combines the provision of sensitivity to the reduced Personal Accomplishment, measures the state of how a person is satisfied with their achievements and competence level. Unlike the other two subscales, low points on this scale indicate Burnout syndrome. The MBI-GS was customized to the characteristics of the population of Georgia, which meant that the tool allowed us to get reliable and valuable information on the target variables, and in the case of a representative selection of the population, the conclusions could be generalized. Reliability indicators of the counted scales of the Georgian version within the original test and our selection (*N* = 168) (internal consistency, Cronbach's alpha coefficient) are presented in Table 2.

**Table 2:** Reliability indicators of the MBI-GS scale.

**Scale**	**Original version of test's Cronbach's α**	**Georgian version of test's Cronbach's α**
Emotional exhaustion	0.90	0.85
Depersonalization	0.79	0.80
Reduced personal accomplishment	0.71	0.80

The three subscales of the MBI-GS including **Emotional Exhaustion** (I feel fatigued when I get up in the morning; I feel I'm working too hard on my job; This work is very stressful for me; I feel I've become more callous toward people), **reduced Personal Accomplishments** (I can easily create a relaxed atmosphere; In my work, I deal with emotional problems very calmly; I have accomplished many worthwhile things in this job; I like to work closely with my colleagues), and **Depersonalization** (I treat some people as if they were impersonal objects; I'm not really interested in what is going on with many of my colleagues) were maintained after the completion of Confirmatory Factor Analysis (CFA) with the following fit indices: χ2 = 247.215, df = 45, *p* = 0.00.

In addition, the set of questions related to satisfaction with the organizational factors, such as **professional growth** (How satisfied are you with professional growth and development in the organization?); **social support** (How satisfied are you with social interactions with colleagues in the organization?); **feedback from the managers** (How satisfied are you with feedback of your manager or supervisor?); **salary** (How satisfied are with your salary?), and **recognition** (How satisfied are you with recognition of your job performance?) were examined with a 5-point Likert scale ranging from “Dissatisfied” to “Satisfied”.

The statistical analysis of the survey data was carried out using IBM SPSS 23 software. The moderation analysis was done using Andrew Hayes's program Process (PROCESS, by Andrew F. Hayes, Procedure for SPSS Release 2.16.1). The statistical analysis of moderation in our study was based on Baron and Kenny's theory (Baron and Kenny, 1986).

## Qualitative data results

Qualitative data were audio-tape recorded, transcribed, and analyzed by the study's investigative team. Recorded interviews were analyzed using a pile sorting technique for identifying themes in qualitative data to identify main organizational characteristics. We used a data-reduction process in which emergent themes were identified and coded to yield a set of core themes.

The qualitative analysis revealed organizational factors such as professional growth and development, social relations at work, feedback from the managers, recognition from the agency and satisfaction with salary. A lack of these factors was identified as the main factor contributing to social workers' burnout and low job satisfaction.

### Professional growth and development

Most of the interviewed social workers mentioned that they needed to improve their professional expertise to work with their beneficiaries. They expressed the need for training in positive parenting, interventions with sexual and other types of violence, and working with adults and children with disabilities and older adults.

“*I lack of skills in dealing with cases that involve sexual violence. I need in-depth trainings in this direction.” (Social Worker, Tbilisi)*

“*I need more competence to work with the families of my beneficiaries.” (Social Worker, Rustavi)*

### Social relations at work

Social workers mentioned that it was important to get assistance from their co-workers in dealing with different cases, especially when they are newly assigned.

“*I always help new social workers. I know how it is hard to work when you do not have on-job trainings. I do it because I know what does it mean to get help when you need it.” (Social Worker, Zugdidi)*

“*We are burned out, we work 24 hours… workload we have is too much, but we help each other and deal with this situation by standing by each other.” (Social Worker, Rustavi)*

However, it needs to be mentioned that social workers also highlighted that supporting peers could be overwhelming and emotionally challenging if it happens in a chaotic and unstructured manner.

“*Social support from my colleagues is important, however, sometimes this is overwhelming as we tend to share all our difficult cases to each other quite often without having any kind of structured format allowing that sharing. We all sit in one office room that we share and when my colleague is talking about his/her challenging case, and seeks to receive some peer support from us, I often feel that this is too much for me, as I have my own cases to deal with and I feel that I am dragged into other's difficulties.”*

It was evident that, though peer social support is important, this should be done in a way that does not compromise social work professional standards and code of ethics. Respecting the confidentiality of the client's personal lives is critical. The use and provision of peer support should be included in the organization's policies and regulated according to professional standards. If done in a chaotic manner, it could contribute to excessive or negative stress and additional emotional pressure.

### Feedback from the managers

Social workers expressed that the supervision they received played a crucial role in their job satisfaction. Reorganization within the State Care Agency that included the formation of a central supervision department improved the quality of work done by social work practitioners. Improvement in the quality of work, in turn, impacted the job satisfaction of social workers.

“*After reorganization supervision is improved. We get intensive feedback from our supervisors. They are involved in every case.” (Social Worker, Rustavi)*

“*My line manager helps me and gives my feedback how to deal with the hard cases and I cannot imagine myself without her support.” (Social Worker, Telavi)*

### Recognition from the agency

In most instances, social workers were not satisfied with their jobs. This was mostly because they did not feel respected and social work was not considered a popular and valued profession. Therefore, social workers looked to the Agency for recognition, which could be in the form of a letter of appreciation, gifts, incentives added to the social worker's wages as a reward for good performance, or health insurance.

“*I know that we do not get normal salary… but at least they can give us a letter of appreciation. It will mean a lot for us.” (Social Worker, Kutaisi)*

“*Our work is not recognized. We do not need much, at least appreciation for what we do.” (Social Workers, Tbilisi)*

### Satisfaction with salary

Most of the interviewed social workers mentioned that their salary was not adequate as it was much less compared to other agencies where social workers were employed.

“*Salary is not similar in different agencies where social workers are employed. And it is reason why there is a high drop outs and transfers to different jobs.” (Social Worker, Kutaisi)*

“*Low salary and a high workload cause a high dropouts from this job.”*

*(Social Worker, Batumi)*

To conclude, all the abovementioned factors can be considered as the main organizational factors that affect social workers' job performance and wellbeing. It impacts employee turnover and the movement of social workers from one job to another, even to different professions, and increases their tendency to quit the job.

## Quantitative data results

### Descriptive data

Before proceeding with the hypotheses testing, frequencies, mean scores, and standard deviations of the main variables were calculated along with bivariate correlations (see Table 3).

**Table 3:** Correlations, means, and standard deviations of main variables.

**Variable**	**1**	**2**	**3**	**4**	**5**	**6**	**7**	**8**	**9**	**10**	**11**	**M**	**SD**
1. Surface acting	–											2.15	2.94
2. Deep acting	0.192^*^	–										3.81	2.79
3. Genuine acting	−0.114	0.515^**^	–									4.48	2.32
4. Emotional exhaustion	0.221^*^	0.119	0.141	–								2.87	2.75
5. Depersonalization	0.356^**^	0.102	−0.104	0.344^**^	–							1.44	1.74
6. Personal accomplishment	−0.131	0.383^**^	0.640^**^	0.080	0.018	–						3.97	2.67
7. Prof. growth	−0.035	0.194^*^	0.301^**^	0.055	−0.025	0.306^**^	–					3.61	1.09
8. Social support	−0.061	0.235^**^	0.298^**^	0.004	0.030	0.438^**^	0.436^**^	–				4.21	0.92
9. Feedback from managers	−0.042	0.208^**^	0.383^**^	0.021	−0.007	0.458^**^	0.515^**^	0.653^**^	–			4.27	0.96
10. Recognition	−0.095	0.087	0.363^**^	−0.154^*^	−0.050	0.436^**^	0.548^**^	0.512^**^	0.565^**^	–		3.83	1.01
11. Satisfaction with salary	−0.088	0.117	0.270^**^	−0.169	−0.077	0.341^**^	0.580^**^	0.398^**^	0.425^**^	0.635^**^	–	3.48	1.01

** *P* < 0.01;

* *P* < 0.05.

For all scales, min = 1 and max = 5.

### Hypothesis testing

To test the predicting nature of variables, hierarchical multiple regression was performed. This method, in particular, through its step-by-step addition of controlling variables, allows us to evaluate each variable's effect on the explanation of dependent variable variation. The variables are centered and measured in the equation according to what contribution the independent variable/variables will add in predicting, for instance, Surface Acting. Demographic variables (gender and region) added to the model are selected based on significant correlation with the dependent variable. The final model is plural, where all the predictor variables are presented. The influence of the independent variables on the dependent variables has been assessed according to standardized beta (β) indicators.

### Direct relationship/predictions

Based on hierarchical multiple regression, the following cause-effect relationship has been outlined between the dimensions of EL and the dimensions of burnout. We estimated the independent variables according to standardized beta (β) indicators.

Emotional exhaustion was predicted by Surface Acting (β = 0.256, *t* = 3.304, *p* < 0.01), and region (β = 0.206, *t* = 2.805, *p* < 0.01). According to the final model, 9% of the variation was explained by this model, F_(2, 165)_ = 8.460, *p* < 0.01.

Depersonalization was predicted by Surface Acting (β = 0.311, *t* = 4.373, *p* < 0.01) and gender (β = 0.257, *t* = 3.612, *p* < 0.01). Approximately 19% of the variation of Depersonalization was explained by this model, F_(2, 165)_ = 19.416, *p* < 0.01.

Personal accomplishment was predicted by Genuine Acting (β = 0.640, *t* = 10.740, *p* < 0.01), and 41% of the variation of Personal Accomplishment was explained by this model, F_(1, 166)_ = 115.348, *p* < 0.01.

Hypothesis H1 was confirmed as Emotional Exhaustion and Depersonalization were predicted by the Surface Acting strategy of Emotional Labor. This meant that social workers who performed EL with the Surface Acting strategy (faking emotions during interaction with the client) were predicted to get Emotional Exhaustion and Depersonalization as work outcomes, both carrying risks for the employees' health.

Hypothesis H3 was confirmed as Personal Accomplishment was a positive outcome and the results showed that it was predicted by the Genuine Acting strategy of EL.

Deep Acting's prediction was not significant. It was tested to determine if EL strategies could predict positive outcomes, like job satisfaction (in the current study sum of satisfaction with organizational factors). General job satisfaction was predicted by Genuine Acting (β = 0.409, *t* = 5.768, *p* < 0.01). Approximately 17% of the variation in job satisfaction was explained by this model, F_(1, 166)_ = 33.275, *p* < 0.01.

### Indirect relationship/moderation

The moderating effects of the organizational factors in the relationship between EL strategies and job burnout expressions (Emotional Exhaustion, Depersonalization, reduced Personal Accomplishments) were analyzed and the statistically significant interaction models are described below.

Testing Hypothesis H2 showed that between Emotional Exhaustion and Surface Acting, and between Depersonalization and Surface Acting, organizational factors did not have significant moderating effects.

However, testing the moderating effects of organizational factors between Genuine Acting and Personal Accomplishment showed that workers who were satisfied with the organizational factors could be expected to experience more Personal Accomplishment than workers who are not satisfied with the organizational factors. The results are as follows:

Regarding the moderation effects between Genuine Acting and Personal Accomplishment, the interaction was significant with the following moderators: **professional growth**; **social support**; **manager's feedback**; **salary**, and **recognition**. The results are presented in Tables 4–8.

**Table 4:** Genuine acting and personal accomplishment, with professional growth as the moderator.

	**Coeff**	**se**	***t***	***p***
Constant	−2.8690	1.8970	−1.5124	0.1324
PA	1.0184	0.0986	10.3323	0.0000
Professi	3.8237	0.6304	6.0654	0000
Int_1	−0.1822	0.0315	−5.7827	0000

Model 1.

R2 = 0.5200; MSE = 2.6234; F = 59.2153.

**Table 5:** Genuine acting and personal accomplishment, with social support as the moderator.

	**Coeff**	**Se**	***t***	***p***
Constant	−1.9624	1.9357	−1.0138	0.3122
PA	1.0487	0.1091	9.6138	0.0000
SocialSu	2.8832	0.5582	5.1655	0.0000
Int_1	−0.1543	0.0293	−5.2745	0.0000

Model 1.

R2 = 0.4959; MSE = 2.7551; F = 52.7736.

**Table 6:** Genuine acting and personal accomplishment, with feedback from the managers as the moderator.

	**Coeff**	**se**	***t***	***p***
Constant	−2.0338	1.9473	−1.0444	0.2978
PA	0.9798	0.1062	9.2253	0.0000
Managers	3.0763	0.5661	5.4338	0.0000
Int_1	−0.1469	0.0286	−5.1301	0.0000

Model 1.

R2 = 0.5004; MSE = 2.7302; F = 54.7617.

**Table 7:** Genuine acting and personal accomplishment, with salary as the moderator.

	**Coeff**	**se**	***t***	***p***
Constant	−2.7535	1.9355	−1.4226	0.1567
PA	1.0388	0.1017	10.2171	0.0000
Salary	3.8184	0.6635	5.7550	0.0000
Int_1	−0.1889	0.0333	−5.6705	0.0000

Model 1.

R2 = 0.5092; MSE = 2.6823; F = 56.7133.

**Table 8:** Genuine acting and personal accomplishment, with recognition as the moderator.

	**Coeff**	**se**	***t***	***p***
Constant	−2.5397	1.9269	−1.3180	0.1893
PA	1.0055	0.1025	9.8091	0.0000
Recognit	3.5734	0.6161	5.7999	0.0000
Int_1	−0.1707	0.0307	−5.5600	0.0000

Model 1.

R2 = 0.5108; MSE = 2.6735; F = 57.0820.

Regarding the moderation effects between Deep Acting and Personal Accomplishment, the interaction was significant with the following moderators: **recognition**; **salary; feedback** from the managers; **social support**; and **professional growth**, and the results are presented in Tables 9–13.

**Table 9:** Deep Acting and Personal Accomplishment, with recognition as the moderator.

	**Coeff**	**se**	***t***	***p***
Constant	−2.1873	2.9269	−0.7473	0.4559
PA	0.9516	0.1557	6.1113	0.0000
Recognit	3.1781	0.9359	3.3959	0.0009
Int_1	−0.1765	0.0466	−3.7854	0.0002

Model 1.

R2 = 0.2227; MSE = 6.1685; F = 15.6638.

**Table 10:** Deep Acting and Personal Accomplishment, with salary as the moderator.

	**Coeff**	**Se**	***t***	***p***
Constant	−1.9565	2.9591	−0.6612	0.5094
PA	0.8938	0.1554	5.7502	0.0000
Salary	3.5478	1.0144	3.4976	0.0006
Int_1	−0.1841	0.0509	−3.6142	0.0004

Model 1.

R2 = 0.2100; MSE = 6.2696; F = 14.5295.

**Table 11:** Deep Acting and Personal Accomplishment, with feedback from the managers as the moderator.

	**Coeff**	**Se**	***t***	***p***
Constant	−1.7600	2.9565	−0.5953	0.5525
PA	0.8655	0.1613	5.3675	0.0000
Managers	3.0089	0.8596	3.5004	0.0006
Int_1	−0.1515	0.0435	−3.4864	0.0006

Model 1.

R2 = 0.2069; MSE = 6.2937; F = 14.2650.

**Table 12:** Deep Acting And Personal Accomplishment, with social support as the moderator.

	**Coeff**	**se**	***t***	***p***
Constant	−1.4814	2.9256	−0.5064	0.6133
PA	0.8455	0.1649	5.1285	0.0000
SocialSu	2.9743	0.8436	3.5256	0.0005
Int_1	−0.1486	0.0442	−3.3603	0.0010

Model 1.

R2 = 0.2070; MSE = 6.2933; F = 14.2688.

**Table 13:** Deep Acting and Personal Accomplishment, with professional growth as the moderator.

	**Coeff**	**se**	***t***	***p***
Constant	−2.2921	2.9166	−0.7859	0.4331
PA	0.8592	0.1515	5.6694	0.0000
Professi	3.7374	0.9692	3.8560	0.0002
Int_1	−0.1793	0.0484	−3.7007	0.0003

Model 1.

R2 = 0.2186; MSE = 6.2012; F = 15.2924.

## Discussion

The findings of the research have supported answering the main questions and, at the same time, raised new questions to be answered in future work. The research aimed to find evidence of the impact of Emotional Labor that social workers perform with clients in terms of their work outcomes, which could be burnout or Personal Accomplishment.

### Surface acting (faking emotions) significantly predicts the negative outcomes of work

An examination of the direct and indirect relationships revealed that Emotional Labor performed with Surface Acting (faking emotions) significantly predicted the negative outcomes of work such as Emotional Exhaustion and Depersonalization. Interestingly, most social workers from the regions^1^ were involved in performing Emotional Labor adopting the Surface Acting strategy and were at risk of Emotional Exhaustion. In rural Georgia, social work practitioners lack the academic knowledge of social work and accordingly are less dedicated to the profession. In fact, in the regions, there are limited human resources who are employed as social workers with a lack of academic education and a formal qualification in social work. Thus, they are less motivated internally to work in the field, which also explains why rural social workers perform their Emotional Labor mostly through Surface Acting. They need further intensive academic training to develop competencies relevant to perform services according to the social work practice standards and code of ethics. In addition, the majority of these workers are in the social work field driven by the need to find employment rather than having a professional passion. Therefore, social workers in these rural areas are at high risk of burnout.

### Genuine acting strategy of Emotional Labor has a direct relationship with personal accomplishment

The Genuine Acting strategy of Emotional Labor, which is a less researched topic in general, had a direct relationship with Personal Accomplishment, which is a positive outcome and supports workers' wellbeing. Moreover, 41% of variances in Personal Accomplishment (the state of how a person is satisfied with his or her achievements and competence level) were explained by Genuine Acting. It appears that, when a social worker's personal attitudes and emotions match with the organization's demands toward clients, then positive outcomes of work, including the wellbeing of personnel, can be expected. Social work is a value-based profession; therefore, genuine accomplishments in social work roles are very critical. Rapport-building with clients, congruence, unconditional positive regard (UPR), accurate empathic understanding, and other principles of humanistic, person-centered counseling approaches (Rogers, 1961) make social workers' jobs extremely emotional, which require conforming to professional ethical standards at the same time. As outlined in the literature, supervision, individual confidential counseling, and guidance on self-care are effective mechanisms to support social workers to deal with Emotional Exhaustion. It is essential to ensure staff has space for reflection and supportive professional relationships so they can think through and plan their response to stressful and complex cases or other aspects of their work. These supportive mechanisms play a vital role in ensuring workers do not experience excessive or negative stress and in preventing burnout. Employers need to advise staff on how to look after themselves physically and mentally to maintain a positive attitude to work and positively manage work tasks and relationships to better manage and prevent stress and maintain a positive work-life balance.

The Deep Acting strategy of Emotional Labor was positively related to Personal Accomplishment but was not significant to predict either negative or positive outcomes.

### Organizational factors have no influence on reducing negative outcomes of EL with surface acting

The findings revealed that organizational factors, such as professional growth, social support, feedback from managers, salary, and recognition have no significant influence on reducing the negative outcomes of Emotional Labor with Surface Acting strategy. It can be explained that a person who performs Emotional Labor with a Surface Acting strategy does not acknowledge the essence of the job position and professional role. These social workers mostly work in rural areas of Georgia and mainly made this career choice, as previously explained, to find employment rather than as a career development opportunity. They have difficulties internalizing social work principles and values. Thus, organizational factors have no power to influence their Surface Acting strategy as they do not fit within the requirements of social work jobs.

What makes someone choose the Surface Acting strategy? What are the antecedents of Surface Acting? According to previous research, employees who had high emotional intelligence, person-job fit, and were in a senior position, were more likely than others, to choose Deep Acting, while individuals with relatively low emotional intelligence, person-job fit, and less experience were more likely than others to follow Surface Acting (Yang and Chen, 2021). This study found the same tendency that social workers with no academic education and/or strong identification with social work values and principles tended to employ a Surface Acting strategy.

### Organizational factors have significant moderation effects on the relationship between Genuine Acting and Personal Accomplishment and between Deep Acting and Personal Accomplishment

Analyzing the indirect effects of the organizational factors between Genuine Acting and Personal Accomplishment, and also Deep Acting and Personal Accomplishment, showed that they had moderate effects. In particular, professional growth, social support, manager's feedback, satisfaction with salary, and recognition had significant moderation effects on the relationships between Genuine Acting and Personal Accomplishment and between Deep Acting and Personal Accomplishment. Thus, it showed that service provider organizations can improve the wellbeing of their workers by considering organizational factors and fostering a positive organizational culture where the staff care about each other, managers are open to giving feedback to their workers, and the administration is eager to encourage workers for good performance. Organizations should also ensure that supportive supervision is provided to social workers to help to deal with the complexity of the casework, individual confidential counseling, and guidance on self-care opportunities, which are available for them to take care of themselves for achieving work-life balance. This study revealed that social workers feel that they work 24/7; however, this is their personal perception, as the study conducted by the GASW in 2022 focused on calculating the time spent during a month on work by the State Care Agency social workers proved the opposite. Social workers' subjective perception that they work 24/7 is explained by the fact that they are emotionally exhausted and burned out because of various factors. These factors stem from issues including the complexity of their work, lack of professional support and supervision, the non-existence of guidance on self-care, limited support services available in the community to empower and assist those in need, low awareness of their mandate by other agencies that they have to work with, and poor working conditions. The qualitative data from this study also supports this finding.

## Limitations

The usage of mixed methods methodology can be considered a strength of our study. Nevertheless, the quantitative research was not free of limitations. The main limitation was its bias stemming from convenience sampling that included only GASW members, limiting the generalizability of the findings.

In addition, the sample mostly consisted of younger adults, primarily women. Additionally, tech-savvy individuals were likely overrepresented. In addition, the qualitative study included different individuals than those who participated in the quantitative study which could be also a problem in terms of triangulation of the obtained data. The cross-sectional design of the current study also has its known drawbacks. Finally, the measure of organizational factors used in the study was not in-depth and included only a few questions.

## Conclusion and practical implications

The results of the research are pragmatic and applicable for social service provider organizations to advance personnel selection and Emotional Labor performance for practitioner social workers to increase effectiveness and gain positive results on the individual as well as on the organizational levels.

The specific findings and recommendations that could be drawn from the study for social service organizations are:

a) The social services sector is a human service, and the core instrument or resource available for vulnerable children and families is the workers themselves, and their knowledge, skills, and values. Social work is demanding. It intensely engages the worker's emotions. It requires thought as well as action to take necessary precautions to proceed ethically when intervening in the lives of others. It requires intellect as well as Emotional Labor. A kind heart, however, is not enough. Professional level and academic training are necessary. Social service work, in short, requires suitably selected and trained workers who are motivated, empathic, reflective, and skilled.
b) The social service sector (e.g., State Care Agency) should further develop and institutionalize worker support systems, such as social work supervision, individual counseling, and guidance on self-care, improve social workers working conditions and management style based on trust and feedback as it is meant as a process to help draw out and build on these qualities and skills of workers and provide emotional and professional support to prevent burnout. All these measures complement each other, and adopting one or two measures alone is not fully sufficient to meet the needs of staff.
c) The discussed forms of support to social workers for emotional management can be partly offered internally by the employing organization. In addition, the support mechanisms should be organized by and offered through various external organizations including a professional association or trade union usually paid for by the employing organization.

## Data availability statement

The original contributions presented in the study are included in the article/supplementary material, further inquiries can be directed to the corresponding author.

## Ethics statement

The studies involving human participants were reviewed and approved by Georgian Association of Social Workers. The patients/participants provided their written informed consent to participate in this study.

## Author contributions

TG participated in developing the study questionnaire, carried out the majority of data processing, analysis and interpretation, and wrote the manuscript. SS conceptualized the research, supported the study planning, developed the study questionnaire, supported the data collection, undertook the data analysis and interpretation, and wrote the manuscript. KG and KL supported the study planning, developed the study questionnaire, supported the data collection, and contributed to the review and editing of the manuscript. SN contributed to the final review of the manuscript. All authors approved the submitted version.

## Footnotes

^1^Demographic data- Employees working in the rural areas of Georgia.
